# 移动LDCT联合AI社区肺结节筛查检出情况及成本分析

**DOI:** 10.3779/j.issn.1009-3419.2026.106.15

**Published:** 2026-06-20

**Authors:** Jianjie WANG, Jun TAO, Meilan ZHOU, Xiaojun WANG, Gang WU, Zhengbin ZHANG, Zhouqin LU, Qinghong LIN, Tiantian WANG, Haozhe ZHANG, Zhiyi ZHENG, Yuehua LI

**Affiliations:** ^1^430030 武汉，武汉市肺科医院结核病控制科（王坚杰，王晓君，张正斌，鲁周琴，王甜甜，张淏喆，李月华）; ^1^Department of Tuberculosis Control; ^2^公共卫生科（陶军，林清宏）; ^2^Department of Public Health; ^3^硚口结核病控制科（周美兰，吴刚，郑志义）; ^3^Qiaokou District Department of Tuberculosis Control, Wuhan Pulmonary Hospital, Wuhan 430030, China

**Keywords:** 肺肿瘤, 移动CT, 社区筛查, 危险因素, 成本效益分析, Lung neoplasms, Mobile CT, Community screening, Risk factors, Cost-effectiveness analysis

## Abstract

**背景与目的:**

肺癌是我国发病率与死亡率最高的恶性肿瘤，低剂量计算机断层扫描（low-dose computed tomography, LDCT）是肺癌早筛的核心手段，但传统固定机构筛查存在基层可及性不足的问题。本研究旨在评估LDCT联合人工智能（artificial intelligence, AI）在武汉城市社区肺结节筛查中的检出特征、影响因素，并开展卫生体系视角下的成本分析，为基层肺结节早期筛查策略优化提供科学依据。

**方法:**

采用基于社区人群的观察性研究设计，于2024年8月至2025年5月对武汉市社区3436例居民进行问卷调查与移动LDCT扫描，招募响应率为26.7%。根据肺部CT筛查报告与数据系统（Lung CT Screening Reporting and Data System, Lung-RADS）对肺结节进行分类，采用多因素*Logistic*回归模型分析临床意义结节（Lung-RADS≥3类）的独立影响因素，通过后退法全变量模型进行敏感性分析，以方差膨胀因子（variance inflation factor, VIF）检验多重共线性；同时遵循CHEERS 2022规范开展描述性成本分析。

**结果:**

筛查人群年龄中位数为63岁，男性占比38.7%，≥60岁人群占比67.3%。总肺结节检出率为84.6%，临床意义肺结节检出率为10.4%，高危肺结节检出率为3.2%。多因素*Logistic*回归显示，60-79岁（OR=2.771, 95%CI: 1.004-7.646, *P*=0.049）、≥80岁（OR=6.224, 95%CI: 1.813-21.369, *P*=0.004）、重度吸烟（OR=1.919, 95%CI: 1.352-2.724, *P*<0.001）、被动吸烟（OR=1.762, 95%CI: 1.200-2.586, *P*=0.004）、接触化学有害物质（OR=2.972, 95%CI: 1.881-4.695, *P*<0.001）、长期厨房油烟暴露（OR=1.411, 95%CI: 1.102-1.807, *P*=0.006）及肺癌家族史（OR=1.866, 95%CI: 1.173-2.967, *P*=0.008）是临床意义肺结节的独立危险因素。项目全周期总成本为666,580元，人均筛查成本为194元。全人群策略下检出单例临床意义肺结节与高危肺结节的成本分别为1857与6060元；针对≥60岁人群筛查可覆盖87.3%的高危肺结节，检出单例高危结节成本降至4676元。

**结论:**

移动LDCT联合AI辅助筛查可有效识别社区肺结节高危个体，老年及多重暴露风险人群是筛查的重点关注对象。本研究提供了该模式的本土化筛查效能参数与成本数据，可为基层肺结节早期筛查策略优化及后续完整卫生经济学评价提供实践依据。

## Abstract (en)

**Background and objective:**

Lung cancer is the malignant tumor with the highest morbidity and mortality in China. Low-dose computed tomography (LDCT) is the core method for early lung cancer screening, but traditional fixed-institution screening has the problem of insufficient accessibility at the grassroots level. This study aims to evaluate the detection characteristics and influencing factors of pulmonary nodules by mobile LDCT combined with artificial intelligence (AI) in urban communities of Wuhan, and conduct a preliminary cost analysis from the health system perspective, so as to provide scientific basis for optimizing early screening strategies for pulmonary nodules at the primary care level.

**Methods:**

A community-based observational study between August 2024 and May 2025 were conducted. A total of 3436 residents in Wuhan communities completed questionnaire surveys and mobile LDCT scans, with a recruitment response rate of 26.7%. Pulmonary nodules were classified according to the Lung CT Screening Reporting and Data System (Lung-RADS). Multivariate *Logistic* regression model was used to analyze the independent influencing factors of clinically significant nodules (Lung-RADS category ≥3). Sensitivity analysis was performed by stepwise backward full-variable model, and multicollinearity was tested by variance inflation factor (VIF). Meanwhile, a descriptive cost analysis was conducted in accordance with the CHEERS 2022 statement.

**Results:**

The median age of the screened population was 63 years, with 38.7% male and 67.3% aged ≥60 years. The overall detection rate of pulmonary nodules was 84.6%, with clinically significant nodules detected in 10.4% and high-risk nodules in 3.2%. Multivariate *Logistic* regression analysis showed that age 60-79 years (OR=2.771, 95%CI: 1.004-7.646, *P*=0.049), age ≥80 years (OR=6.224, 95%CI: 1.813-21.369, *P*=0.004), heavy smoking (OR=1.919, 95%CI: 1.352-2.724, *P*<0.001), secondhand smoke exposure (OR=1.762, 95%CI: 1.200-2.586, *P*=0.004), exposure to harmful chemicals (OR=2.972, 95%CI: 1.881-4.695, *P*<0.001), long-term kitchen oil fume exposure (OR=1.411, 95%CI: 1.102-1.807, *P*=0.006), and family history of lung cancer (OR=1.866, 95%CI: 1.173-2.967, *P*=0.008) were independent risk factors for clinically significant nodules. The total cost of the project was 666,580 CNY, with an average screening cost of 194 CNY per person. Under the whole population strategy, the costs for detecting one clinically significant nodule and one high-risk nodule were 1857 and 6060 CNY, respectively. Screening targeted at individuals aged ≥60 years could cover 87.3% of high-risk nodules, with the cost per high-risk nodule detected reduced to 4676 CNY.

**Conclusion:**

Mobile LDCT combined with AI-assisted screening can effectively identify high-risk individuals of pulmonary nodules in community settings. The elderly and people with multiple exposure risks should be the priority population for screening. This study provides localized screening efficiency parameters and cost data of this model, which can provide practical basis for the optimization of primary pulmonary nodule early screening strategies and subsequent complete health economic evaluation.

肺癌是全球发病率和死亡率最高的恶性肿瘤，严重威胁居民健康并造成沉重疾病负担^[1,2]^。肺结节是肺癌的早期影像学表现，肺部计算机断层扫描（computed tomography, CT）筛查报告和数据系统（Lung CT Screening Reporting and Data System, Lung-RADS）有助于识别高危人群并指导后续诊疗。目前低剂量CT（low-dose CT, LDCT）是目前国际公认最有效的肺癌早期筛查手段。美国国家肺癌筛查试验（National Lung Screening Trial, NLST）及荷兰-比利时随机化肺癌筛查试验（Dutch-Belgian Randomized Lung Cancer Screening Trial, NELSON）试验^[3-5]^均已证实，LDCT筛查可显著降低高危人群肺癌死亡率，降幅达20%-24%。然而，传统LDCT筛查依赖固定医疗机构的设备配置，在基层社区、偏远地区及行动不便人群中普及率较低，存在显著的地域与资源可及性壁垒^[6]^。近年来，移动CT筛查单元（mobile CT unit）的兴起为解决这一难题提供了新路径。该模式将高分辨率影像设备直接下沉至社区，已在国内外研究^[7,8]^中初步证实其可行性。同时，人工智能（artificial intelligence, AI）辅助诊断系统的快速发展为大规模人群筛查提供了技术支撑。研究^[9]^显示，AI辅助阅片可显著提升肺结节检出敏感性，尤其对于低年资医师，敏感性提升幅度可达25%以上。AI可自动检测肺结节、进行风险分级，显著提升阅片效率与诊断一致性，尤其适用于资源匮乏地区的筛查场景^[8]^。

系统分析人群肺结节检出特征及其影响因素，可为优化肺癌早筛策略、实现精准分层管理提供直接依据。目前我国关于移动CT联合AI在普通社区居民中开展肺结节筛查的系统性研究仍较缺乏，尤其是在影响因素识别与卫生经济学评价方面尚缺乏高质量的实证数据。本研究基于武汉市社区的移动CT筛查项目，构建“AI初筛-医师阅片-专家审核-多学科会诊”协同诊断模式，分析肺结节检出特征及影响因素，并初步评估筛查成本效益，为优化社区肺结节筛查与风险分层能力提供科学依据。

## 1 资料与方法

### 1.1 研究对象

本研究为基于社区人群的观察性研究，于2024年8月至2025年5月在武汉市社区开展。通过武汉市目标社区居委会开展线下宣传与线上通知，共覆盖社区常住居民12,876例，最终完成知情同意并纳入研究的有效样本为3436例，招募响应率约26.7%。纳入标准：（1）年龄≥18岁；（2）为本社区常住居民；（3）无精神或认知障碍，能配合完成调查与检查。排除标准：（1）既往有肺癌或其他恶性肿瘤病史；（2）妊娠或计划妊娠妇女；（3）3个月内接受过胸部CT检查者。本研究已通过武汉市肺科医院伦理委员会审查[202(4)]年临审第（017）号。

### 1.2 调查方法和工具

采用问卷调查与LDCT相结合的筛查方式。问卷内容包括人口学特征、吸烟史、职业暴露史、家族史、生活环境、肺部疾病及呼吸道症状等。吸烟总量定义为包年，计算公式为每日吸烟包数×吸烟年数。被动吸烟定义为每周至少1天暴露于他人吸烟环境>15 min。厨房油烟暴露根据自我报告分为“长期”（每日烹饪>30 min，持续≥10年）、“短期”（不符合长期标准）和“无”。

### 1.3 筛查流程与质量控制

本研究构建了“社区动员-移动CT采集-AI辅助初筛-远程阅片-专家审核-多学科会诊”六位一体的筛查路径。移动CT车进驻社区后，由经统一培训的调查员完成问卷访谈与知情同意。LDCT影像通过5G网络实时传输至云端影像归档和通信系统（Picture Archiving and Communication Systems, PACS）。所有LDCT影像首先经AI辅助诊断系统（InferRead CT Lung，北京推想科技）进行初筛与结节自动标记，完成结节自动检出、大小/密度精准测量、恶性风险初分级，为双阅片医师提供诊断参考。随后，LDCT影像由2名高年资放射科医师独立线上阅片，结合AI标记结果完成Lung-RADS分类^[10]^。若两者分类不一致，提交第3位影像专家仲裁。对于Lung-RADS≥4类或临床高度怀疑恶性者，邀请胸外科、呼吸科、肿瘤科等多学科专家进行集中讨论，制定个体化诊疗方案。数据采用双录入核查，确保一致性。

### 1.4 统计学方法

采用SPSS 26.0进行数据分析。年龄构成经*Kolmogorov-Smirnov*检验呈非正态分布，以中位数和四分位数（Q1, Q3）描述；计数资料以例数和百分比表示，组间比较采用卡方检验。将单因素分析中具有统计学意义的变量纳入多因素*Logistic*回归模型，分别采用逐步向前法（纳入标准α=0.05，剔除标准α=0.10）与后退法构建全变量模型，开展敏感性分析验证结果稳健性；采用方差膨胀因子（variance inflation factor, VIF）检验变量间多重共线性，VIF<5为无显著多重共线性。*P*<0.05为差异有统计学意义。

### 1.5 成本分析框架

本研究为卫生体系视角下的描述性成本分析，旨在为后续完整经济学评价提供基础参数，报告遵循CHEERS 2022清单规范^[11]^。成本测算采用基于活动的成本核算法（activity-based costing, ABC），按社区宣传、移动CT采集、AI初筛、远程阅片、专家审核及多学科会诊全流程逐环节归集。成本分为直接医疗成本与直接非医疗成本：直接医疗成本包括移动CT车租赁与维护、CT耗材、AI诊断系统使用费、影像存储与传输费用；直接非医疗成本包括社区宣传发动费及项目工作人员人力成本（按《武汉统计年鉴2025》^[12]^卫生行业平均工资折算）。未计入患者时间、交通等广义间接成本。固定成本按实际筛查人数均摊，宣传费按项目总支出计入。本研究以每检出1例临床意义结节（Lung-RADS≥3类）成本和每检出1例高危结节（Lung-RADS 4类）成本为中间结局指标，计算例均检出成本。

## 2 结果

### 2.1 筛查人群基本情况

本研究共筛查3436例调查对象，其中男性1331例（38.7%），女性2105例（61.3%）。年龄范围为21-92岁，中位数为63（四分位数：55，70）岁。与2024年武汉市户籍人口构成^[12]^相比，筛查人群女性比例偏高（61.3% *vs* 49.6%），≥60岁人群占比显著偏高（67.3% *vs* 24.1%），而18-39岁青壮年人群占比明显偏低（2.7% *vs* 29.3%）。当前不吸烟者2631例（76.6%），当前吸烟者805例（23.4%）。被动吸烟者2941例（85.6%），接触化学有害物质者117例（3.4%），长期厨房油烟暴露者2294例（66.8%），有肺癌家族史者156例（4.5%）。

### 2.2 基于Lung-RADS的结节检出分布

3436例调查对象中，Lung-RADS分类阴性530例（15.4%），1级35例（1.0%），2级2512例（73.1%），3级249例（7.2%），4A级60例（1.7%），4B级27例（0.8%），4X级23例（0.7%）。总结节（1-4X类）检出率为84.6%（2906/3436），临床意义结节（≥3类）检出率为10.4%（359/3436），高危结节（4类）检出率为3.2%（110/3436）。

### 2.3 临床意义肺结节的风险因素分析

单因素分析（表1）显示，年龄、吸烟总量、被动吸烟、接触化学有害物质、厨房油烟暴露及肺癌家族史与临床意义结节（≥3类）有显著关联（均*P*<0.05），而性别、肺部疾病史及呼吸道症状与临床意义结节（≥3类）无显著关系。

**表1:** 临床意义结节组与非临床意义结节组的人群特征分布构成对比

Variable	Total (*n*=3436)	Clinically significant nodule group (*n*=359)			Non-clinically significant nodule group (*n*=3077)		*χ* ^2^	*P*
		*n*	%		*n*	%		
Gender							0.462	0.497
Male	1331	145	10.9		1186	89.1		
Female	2105	214	10.2		1891	89.8		
Age (yr)							76.197	<0.001
20-39	94	4	4.3		90	95.7		
40-59	1028	42	4.1		986	95.9		
60-79	2271	303	13.3		1968	86.7		
≥80	43	10	23.3		33	76.7		
Smoking amount (pack-years)							21.623	<0.001
Never smoke	2631	245	9.3		2386	90.7		
Mild (≤10)	134	14	10.4		120	89.6		
Moderate (11-30)	393	51	13.0		342	87.0		
Severe (>30)	278	49	17.6		229	82.4		
Secondhand smoke exposure							7.051	0.008
Yes	2941	324	11.0		2617	89.0		
No	495	35	7.1		460	92.9		
Exposure to harmful chemicals							36.983	<0.001
Yes	117	32	27.4		85	72.6		
No	3319	327	9.8		2992	90.2		
Kitchen oil fume exposure							11.186	0.004
Long-term	2294	212	9.2		2082	91.8		
Short-term	199	23	12.4		176	87.6		
No	943	124	13.2		819	86.8		
Lung disease history							0.771	0.380
Yes	266	32	12.0		234	88.0		
No	3170	327	10.3		2843	89.7		
Family history of lung cancer							5.433	0.020
Yes	156	25	16.0		131	84.0		
No	3280	334	10.2		2946	89.8		
Respiratory symptoms							0.001	0.975
Yes	257	27	10.5		230	89.5		
No	3179	332	10.4		2847	89.6		

Row percentages are shown, representing the detection rates of clinically significant and non-clinically significant nodules in each subgroup.

### 2.4 影响Lung-RADS≥3类结节的多因素*Logistic*回归分析

将单因素分析中*P*<0.05的变量纳入多因素*Logistic*回归模型，结果（表2）显示：60-79岁（OR=2.771, 95%CI: 1.004-7.646）、≥80岁（OR=6.224, 95%CI: 1.813-21.369）、重度吸烟（>30包年：OR=1.919, 95%CI: 1.352-2.724）、被动吸烟（OR=1.762, 95%CI: 1.200-2.586）、接触化学有害物质（OR=2.972, 95%CI: 1.881-4.695）、长期厨房油烟暴露（OR=1.411, 95%CI: 1.102-1.807）及肺癌家族史（OR=1.866, 95%CI: 1.173-2.967）是临床意义结节的独立风险因素（均*P*<0.05）。为验证变量筛选的稳健性，进一步采用后退法（剔除标准*α*=0.10）进行全变量模型分析，各变量OR值及95%CI方向与逐步向前法一致，结果稳定。各变量VIF范围为1.004-1.406，无显著多重共线性。

**表2:** 临床意义肺结节影响因素的多因素Logistic回归分析结果

Variable	*β*	*SE*	Wald *χ*^2^	*P*	OR (95%CI)
Age (yr)					
20-39					1.000 (Reference)
40-59	-0.194	0.537	0.131	0.717	0.823 (0.287-2.358)
60-79	1.019	0.518	3.871	0.049	2.771 (1.004-7.646)
≥80	1.828	0.629	8.442	0.004	6.224 (1.813-21.369)
Smoking amount					
Never					1.000 (Reference)
Mild (≤10 pack-years)	0.215	0.298	0.522	0.470	1.240 (0.692-2.225)
Moderate (11-30 pack-years)	0.303	0.170	3.146	0.076	1.352 (0.969-1.887)
Severe (>30 pack-years)	0.652	0.179	13.315	<0.001	1.919 (1.352-2.724)
Secondhand smoke exposure					
No					1.000 (Reference)
Yes	0.566	0.196	8.371	0.004	1.762 (1.200-2.586)
Exposure to harmful chemicals					
No					1.000 (Reference)
Yes	1.089	0.233	21.791	<0.001	2.972 (1.881-4.695)
Kitchen oil fume exposure					
No					1.000 (Reference)
Short-term	0.292	0.250	1.366	0.243	1.339 (0.821-2.186)
Long-term	0.344	0.126	7.466	0.006	1.411 (1.102-1.807)
Family history of lung cancer					
No					1.000 (Reference)
Yes	0.624	0.237	6.939	0.008	1.866 (1.173-2.967)

OR: odds ratio; CI: confidence interval; SE: standard error.

### 2.5 筛查策略的成本构成情况

本次移动CT社区筛查总成本为666,580元（表3），平均每例筛查成本194元（含AI辅助诊断及人员成本）。全人群筛查策略下，检出1例临床意义结节（≥3类）的成本为1857元，检出1例高危结节（4类）的成本为6060元。若将筛查对象限定为≥60岁人群（占筛查总人口的67.3%），可覆盖87.3%（96/110）的高危结节，检出单例高危结节成本降至4676元，较全人群策略降低22.8%（表4）。

**表3:** 移动CT社区筛查项目成本构成表

Cost category	Cost details	Total cost (CNY)	Calculation basis and description
Direct medical cost	Mobile CT car rental and maintenance and AI-assisted diagnosis system usage fee	570,000	Allocated linearly according to the total number of screened participants
	CT scanning consumables	1380	Calculated according to actual consumption
Direct non-medical cost	Project staff labor cost	81,200	Converted according to the annual average wage of health industry in Wuhan Statistical Yearbook 2025^[12]^
	Community publicity and mobilization fee	14,000	Total project expenditure
Total cost		666,580	

CT: computed tomography; AI: artificial intelligence.

**表4:** 不同筛查人群策略的单位检出成本对比表

Screening strategy	Number of covered participants	Coverage rate of high-risk nodules (%)	Total cost (CNY)	Cost per capita (CNY)	Cost per detected clinically significant nodule (CNY)	Cost per detected high-risk nodule (CNY)
Whole population strategy (≥18 yr)	3436	100.0	666,580	194	1857	6060
Age-stratified strategy (≥60 yr)	2314	87.3	448,916	194	1634	4676

## 3 讨论

### 3.1 移动CT筛查人群检出特征分析

本研究首次在武汉城市社区层面系统性评估了移动LDCT联合AI辅助筛查肺结节的可行性与检出效能。结果显示肺结节检出率为84.6%，临床意义结节（Lung-RADS≥3类）检出率为10.4%，高危结节（4类）检出率为3.2%。与Pua等^[7]^和Tao等^[8]^相比，本研究检出水平介于两者之间，但被动吸烟率显著高于上述两项研究，而主动吸烟率则较低，可能与本研究中女性比例较高有关，提示不同地域、性别结构和生活方式背景下肺结节检出谱存在异质性。与徐国厚等^[13]^基于体检人群的研究相比，本研究肺结节检出率略高，可能与移动CT模式覆盖了更多高龄及行动不便人群，且采用1 mm薄层重建联合AI辅助初筛提高了微小结节的检出敏感性有关。但高检出率也伴随假阳性风险增加，如何在筛查实践中平衡敏感度与特异度，仍是LDCT筛查需持续优化的问题^[14]^。

### 3.2 临床意义肺结节的危险因素及其公共卫生意义

多因素Logistic回归分析显示，年龄≥60岁、重度吸烟（>30包年）、被动吸烟、接触化学有害物质、长期厨房油烟暴露及肺癌家族史是Lung-RADS≥3类肺结节的独立危险因素。单因素分析中长期厨房油烟暴露组的粗检出率（9.2%）反而低于无暴露组（13.2%），主要是因为无暴露组中高龄、重度吸烟等混杂因素比例更高所致的负向混杂；经多因素校正后，长期油烟暴露的独立危险效应得以显现（OR=1.411, 95%CI: 1.102-1.807）。上述发现与国内外多项研究^[15,16]^一致。其中，长期厨房油烟暴露（OR=1.411）是具有显著本土特征的独立危险因素。与我国传统烹饪方式中高温煎炒、油烟排放不当明显关联，油烟中含有多环芳烃、苯并芘等明确致癌物^[17]^。该结果提示，在制定我国肺癌高危人群筛选标准时，应系统纳入烹饪油烟暴露、被动吸烟等环境行为因素。接触化学有害物质（OR=2.972）的风险效应较为显著，表明职业健康监护在肺癌一级预防中的重要地位。国际癌症研究机构已将多种职业暴露列为肺癌确证致癌物^[18]^。建议在社区健康教育中加强厨房通风、正确使用抽油烟机的宣传，并将其纳入肺癌一级预防的核心内容。

### 3.3 无症状筛查的必要性与筛查策略优化

本研究中92.5%临床意义肺结节检出者在筛查前无任何呼吸道症状，并且有无症状组检出率差异无统计学意义，与Aberle等^[4]^的研究结论一致，提示依赖症状识别肺癌高危个体存在严重滞后性。国际指南^[10,19]^已明确提出，肺癌筛查应基于年龄、吸烟、家族史、职业暴露等多维风险评分，而非症状存在与否。本研究结果进一步支持在社区层面推广基于风险分层的精准筛查策略。

### 3.4 成本分析的结果解读与研究价值

本研究基于真实世界筛查数据开展卫生体系视角下的描述性成本分析，结果显示移动LDCT联合AI辅助筛查人均成本为194元，检出单例临床意义肺结节及高危结节的成本分别为1857与6060元。若将目标人群聚焦于≥60岁居民，可覆盖87.3%的高危结节，检出单例高危结节成本为4676元，投入产出比显著提升。与国内同类研究相比，本项目的筛查成本具有一定优势。Huang等^[20]^2025年在福建农村开展的移动CT+AI筛查研究中，人均筛查成本为118.47元，检出单例疑似肺癌的成本为11,143.56元。本研究人均成本略高于福建研究，可能与武汉地区人力成本较高有关；但高危结节检出成本低于福建研究，提示本研究的筛查人群选择及风险分层策略可能更具效率。徐欢欢等^[21]^的研究也表明，AI辅助阅片用于早期肺癌筛查具有良好的卫生经济学价值。与部分城市固定机构LDCT筛查项目（250-400元/人）^[22,23]^相比，移动LDCT模式在资源下沉与规模化应用中展现出成本优势。AI辅助诊断系统的引入已被证实可显著提升肺小结节检出率、缩短阅片时间、降低漏诊率。卫生技术评估系统评价^[9]^显示，AI辅助可将放射科医师的结节检出敏感性提高15%-25%，且对低年资医师的提升效果更为显著。

本分析的核心价值在于提供了武汉城市社区移动LDCT+AI筛查的本土化成本参数与执行效率数据，可为后续开展包含对照策略、增量分析及长期健康结局的完整卫生经济学评价奠定基础。由于本研究仅采用结节检出相关中间结局指标，未设置平行对照、未纳入生存获益或质量调整生命年（quality-adjusted life year, QALY）等最终健康产出，尚不能判定该模式具有成本效果优势，其长期卫生经济学价值仍需依托本队列开展长期随访，并通过 Markov模型等方法进一步系统评估^[18]^。

### 3.5 本研究的局限性

包括以下几方面：（1）本研究为社区自愿招募的横断面观察性研究，存在选择偏倚，筛查人群年龄偏大（≥60岁占67.3%）、女性偏多（61.3%），与武汉市常住人口构成存在明显差异，且招募响应率仅26.7%，未参与者特征未知，结果外推至全人群需谨慎，此外，年龄分层中20-39岁（*n*=94）与≥80岁（*n*=43）样本量有限，致其OR估计值CI较宽，相关结果应谨慎解读；（2）因AI系统涉及商业版权与算法保护，无法开展AI与人工阅片的一致性检验及诊断效能量化分析，未能直接评估AI的独立辅助价值；（3）卫生经济学部分仅为卫生体系视角下的描述性成本分析，未设置对照、未纳入长期健康结局，不构成完整经济学评价，且未开展卫生经济学敏感性分析，结果稳健性有限，仅可为后续研究提供基础参数。

综上，移动LDCT联合AI辅助筛查可提升基层肺结节早期筛查与风险分层能力，基于多维度风险因素的精准分层策略有助于优化资源配置。建议在后续工作中整合AI辅助诊断系统，开展长期随访与完整成本-效用分析，并将移动CT+AI筛查模式纳入基层慢性病防控体系，优先覆盖老年及多暴露风险人群。
